# Preferential effect of isoflurane on top-down vs. bottom-up pathways in sensory cortex

**DOI:** 10.3389/fnsys.2014.00191

**Published:** 2014-10-07

**Authors:** Aeyal Raz, Sean M. Grady, Bryan M. Krause, Daniel J. Uhlrich, Karen A. Manning, Matthew I. Banks

**Affiliations:** ^1^Department of Anesthesiology, School of Medicine and Public Health, University of WisconsinMadison, WI, USA; ^2^Department of Anesthesiology, Rabin Medical Center, Petah-Tikva, Israel, Affiliated with Sackler School of Medicine, Tel Aviv UniversityTel Aviv, Israel; ^3^Neuroscience Training Program, University of WisconsinMadison, WI, USA; ^4^Department of Neuroscience, University of WisconsinMadison, WI, USA

**Keywords:** cortical column, anesthesia, auditory evoked response, neocortex, multimodal integration, current source density

## Abstract

The mechanism of loss of consciousness (LOC) under anesthesia is unknown. Because consciousness depends on activity in the cortico-thalamic network, anesthetic actions on this network are likely critical for LOC. Competing theories stress the importance of anesthetic actions on bottom-up “core” thalamo-cortical (TC) vs. top-down cortico-cortical (CC) and matrix TC connections. We tested these models using laminar recordings in rat auditory cortex *in vivo* and murine brain slices. We selectively activated bottom-up vs. top-down afferent pathways using sensory stimuli *in vivo* and electrical stimulation in brain slices, and compared effects of isoflurane on responses evoked via the two pathways. Auditory stimuli *in vivo* and core TC afferent stimulation in brain slices evoked short latency current sinks in middle layers, consistent with activation of core TC afferents. By contrast, visual stimuli *in vivo* and stimulation of CC and matrix TC afferents in brain slices evoked responses mainly in superficial and deep layers, consistent with projection patterns of top-down afferents that carry visual information to auditory cortex. Responses to auditory stimuli *in vivo* and core TC afferents in brain slices were significantly less affected by isoflurane compared to responses triggered by visual stimuli *in vivo* and CC/matrix TC afferents in slices. At a just-hypnotic dose *in vivo*, auditory responses were enhanced by isoflurane, whereas visual responses were dramatically reduced. At a comparable concentration in slices, isoflurane suppressed both core TC and CC/matrix TC responses, but the effect on the latter responses was far greater than on core TC responses, indicating that at least part of the differential effects observed *in vivo* were due to local actions of isoflurane in auditory cortex. These data support a model in which disruption of top-down connectivity contributes to anesthesia-induced LOC, and have implications for understanding the neural basis of consciousness.

## Introduction

Although in widespread use for >150 years, how anesthetics cause loss of consciousness (LOC) remains one of the great unsolved mysteries in biomedical science. Elucidating these mechanisms would benefit patient care in terms of improved monitoring and more selective anesthetic agents, and would provide insight into neural mechanisms of consciousness. Indeed, in recent years, research in the fields of anesthetic mechanisms and the neural basis of consciousness have begun to converge (Mashour, 2006; Alkire et al., 2008b; Shushruth, 2013).

We have extensive knowledge of the molecular targets and behavioral effects of anesthetic agents (Antkowiak, 2001; Rudolph and Antkowiak, 2004; Franks, 2008). Much less is known about how anesthetics act at the level of cortical circuits. Previous studies focused on the dramatic reduction in cortical activity observed at surgical anesthetic doses (Schwender et al., 1993a), and imaging and electrophysiological studies in thalamus suggested that anesthetics suppress ascending information flow into cortex (Ries and Puil, 1999; Alkire et al., 2000; Schroter et al., 2012). Because TC information transfer has been hypothesized as the key mediator for consciousness (Llinas et al., 1998), these observations formed the basis of the *thalamic switch hypothesis* of anesthetic-induced LOC (Alkire et al., 2000). However, studies have also shown that suppression of cortical sensory responses by anesthetics can be unrelated to awareness (Dueck et al., 2005; Kerssens et al., 2005; Plourde et al., 2006), that sensory evoked responses can even be enhanced dramatically under anesthesia compared to waking conditions (Imas et al., 2005b), and that anesthetics selectively suppress “matrix” thalamic nuclei, which provide largely modulatory TC input, compared to “core” thalamic nuclei, which provide largely driving TC input (Jones, 1998; Liu et al., 2013; Saalmann, 2014). Thus, evidence suggests that during anesthesia-induced LOC, as during sleep, external sensory stimuli activate cortex but fail to become incorporated into the hierarchical processing stream (Liu et al., 2011; Hobson and Friston, 2012). These data have motivated an alternative hypothesis, which we call here the *cortico-thalamic network disruption hypothesis* that emphasizes anesthetic effects on CC connectivity and information processing. This hypothesis derives from two related theories. The first, the *information integration theory of consciousness*, proposes that consciousness relies on the dense interconnectivity within the TC network and the vast number of possible network states (Tononi, 2004). According to this hypothesis, anesthetics act across wide areas of cortex to reduce the repertoire of network states (i.e., information) and connectivity (i.e., integration) (Alkire et al., 2008b). In the other, the *cognitive unbinding hypothesis*, anesthetics disrupt the cortical integration of sensory information to prevent a unified percept of the external world (Mashour, 2013).

Specific ideas about which connections are targeted under the *cortico-thalamic network disruption hypothesis* have emerged recently, based on predictive coding models of neocortex. These models posit comparisons of observed, bottom-up sensory information with top-down predictions based on memory and context, all simultaneously at multiple hierarchical processing stages (Grossberg and Versace, 2008; Bar, 2009; George and Hawkins, 2009; Bastos et al., 2012). Processes such as priming, context, expectation, and attention influence responses to sensory stimuli (Warren, 1970; Haist et al., 2001; Alain and Izenberg, 2003; Alain, 2007; Davis and Johnsrude, 2007; Fritz et al., 2007; Todorovic et al., 2011; Chennu et al., 2013; Kok et al., 2013), likely via modulation of infra- and supragranular pyramidal cells due to the concentration of descending CC and “matrix” TC (see below) inputs to these layers (Zeki and Shipp, 1988; Felleman and Van Essen, 1991; Cauller, 1995). This comparison or integration of bottom-up and top-down information streams is postulated to be a critical component of sensory awareness, and its disruption is thought to represent a common mechanism for LOC in natural and clinically relevant conditions. Thus, several lines of evidence suggest that LOC due to anesthesia and slow wave sleep and in patients in vegetative states is caused by suppressed CC connectivity and thus disruption of this integrative process. During midazolam-induced LOC and during slow-wave sleep, local cortical responses to transcranial magnetic stimulation are enhanced locally but the spread of activity due to CC interactions is reduced (Massimini et al., 2005; Ferrarelli et al., 2010). Furthermore, under a variety of anesthetic regimes, long range descending CC connectivity is preferentially suppressed (Imas et al., 2005a; Peltier et al., 2005; Alkire, 2008; Lee et al., 2009, 2013a,b; Ku et al., 2011; Liu et al., 2011; Schrouff et al., 2011; Boly et al., 2012; Jordan et al., 2013; Blain-Moraes et al., 2014; Mashour, 2014). Similar results demonstrating selective loss of descending CC connectivity were demonstrated in vegetative states as well (Boly et al., 2011). Finally, general anesthetics eliminate contextual modulation of responses in primary visual cortex that are likely mediated by top-down connections, but leave bottom-up responses intact (Lamme et al., 1998) and suppress integration of local receptive field information (Pack et al., 2001). However, in none of these studies were effects of anesthetics on bottom-up vs. top-down projections tested directly. Many of these studies are based on EEG methods, which are unable to measure thalamic activity, leaving the thalamic involvement in this process as a theoretical consideration rather than actual measurement. Even fMRI studies often lack the spatial resolution to differentiate between anesthetic effects on core vs. matrix thalamic nuclei.

Sensory cortex in general, and auditory cortex specifically, is a useful system to test these competing hypotheses about anesthesia-induced LOC (Imas et al., 2004; Banks, 2010; Liu et al., 2011). This area is relevant to clinical monitoring of anesthesia depth (Drummond, 2000) and for evaluating modulation of sensory information received by the brain. It is possible to activate selectively different projection pathways. Ascending (bottom-up) afferents from ventral medial geniculate (MGv; “core TC afferents”) terminate with highest density in layers 3 and 4 of auditory cortex (Scheel, 1988; Roger and Arnault, 1989; Romanski and Ledoux, 1993; Winer et al., 1999; Polley et al., 2007; Storace et al., 2010; Smith et al., 2012), and their activation via acoustic stimuli *in vivo* leads to a stereotypical synaptic response in these layers (Kaur et al., 2005; Szymanski et al., 2009). Other inputs arising from descending CC afferents as well as other thalamic nuclei (e.g., medial division of MG; “matrix TC afferents”) also provide large numbers of synaptic connections (Rockland and Virga, 1989; Salin et al., 1995; Budd, 1998) and are likely to modulate responses to ascending input (Sandell and Schiller, 1982) and regulate information transmission (Saalmann, 2014), in some cases driving columnar activity prior to or in the absence of ascending input (Cauller and Kulics, 1991; Mignard and Malpeli, 1991; Krupa et al., 2004). Although these descending CC and matrix TC afferents likely serve distinct functions, for simplicity and for the purposes of this study we will refer to these afferents as top-down due to their largely modulatory nature and the overlap in their projection patterns. These projections target preferentially layers 1, 2, 5, and 6 (Shi and Cassell, 1997; Kimura et al., 2004; Smith et al., 2012), and their activation will thus lead to a response pattern distinct from core TC afferents. We, and others, have shown that visual responses in auditory cortex are carried by descending cortical and matrix thalamic afferents (Budinger et al., 2006; Bizley et al., 2007; Smith et al., 2010; Banks et al., 2011), and thus visual stimuli will activate top-down pathways in auditory cortex *in vivo*. In brain slices, CC and matrix TC pathways can be activated directly by electrical stimulation.

In this paper, we used electrophysiological recordings from whole columns *in vivo* and *in vitro* and activated ascending thalamic and descending cortical pathways selectively to test the hypothesis of a differential effect of anesthetics on ascending vs. descending pathways.

## Materials and methods

All procedures followed the NIH Guide for the Care and Use of Laboratory Animals and were in accordance with institutional guidelines.

### *In vivo* experiments

#### Electrode implantation

Female Harlan Sprague Dawley (*n* = 2) or ACI (*n* = 8) rats (170–250 gm) were housed individually in transparent Plexiglas cages in dedicated rooms (12:12 reversed light-dark cycle, on at 6 p.m., 23 ± 1°C; food and water *ad libitum*). Animals were chronically implanted under aseptic conditions with 1 × 16 single shank silicon electrode arrays (15 μm thick, 150 μm wide) with iridium recording sites (177 μm^2^; 1.5 MΩ; Neuronexus Technologies, Ann Arbor, MI). Anesthesia was induced and maintained with isoflurane (1.5–2% in 50% O_2_/50% room air). Meloxicam (1 mg/kg SQ) was administered during surgery to manage pain and swelling. Rats were kept on an infrared heating pad throughout surgery and recovery to maintain core temperature at 37 ± 0.5°C. Core auditory cortex was located stereotaxically (Doron et al., 2002; Polley et al., 2007) and electrode placement confirmed *post-hoc* histologically (see below). A craniotomy ~2.5 mm^2^ was made over left auditory cortex using a surgical drill and an ultra-fine burr bit and the dura dissected. The electrode was advanced at an angle normal to the surface of the brain until the most superficial recording site was embedded just below the pial surface. Ground and reference electrodes were attached to skull screws placed over the contralateral parietal cortex and over the cerebellum. The craniotomy was sealed with silicone elastomer (Kwik Sil, World Precision Instruments, Sarasota, FL) and the electrode array was fixed to the skull screws and the skull with dental acrylic. Connectors that served as mounting devices for head-mounted LEDs were fixed to the skull using dental acrylic. Animals were medicated postoperatively for pain (buprenorphine 0.05 mg/kg SQ and meloxicam 1 mg/kg SQ) and monitored daily for signs of discomfort and infection. The animals were allowed to recover for 1 week before their first recording session.

#### Electrophysiological recordings

Recordings were performed in a sound-proof chamber (Industrial Acoustics Company, Inc., Bronx, NY), inside which animals were placed in a home-made gas-tight acrylic enclosure (20 × 19 × 11 cm) that had gas inflow and outflow ports for administering and scavenging isoflurane and a gas sampling port for monitoring the isoflurane concentration using a commercial monitor (Multigas Monitor 602, Criticare Systems, Waukesha, WI). A heating pad was placed in the bottom of the enclosure to keep the animals warm during anesthesia application. A small speaker (TDT-ES1, Tucker Davis Technologies, Alachua, FL) was mounted inside the enclosure, oriented toward the animal. The speaker was calibrated using a microphone (#4016, ACO Pacific, Inc., Belmont, CA) placed approximately 4 cm from the speaker, and stimuli presented at approximately 20–80 dB SPL assuming the animal's head was this distance from the speaker. Since the animal was unrestrained, actual stimulus levels on each trial varied slightly. Speaker output varies by < ±10 dB SPL over the range 4–60 kHz. Free-field stimuli were applied using commercial software (Brainware, RPVDX, Tucker-Davis Technologies, Alachua, FL) and custom software written in Matlab. A 16 channel headstage (TDT RA16) on a flexible tether was plugged into an Omnetics connector on the animal's head. For all electrophysiological recordings, responses were bandpass-filtered at 2–7500 Hz, amplified 5000–10,000 ×, digitized at 24.414 kHz (TDT RZ5 or RX5) and collected using Brainware. Local field potentials (LFPs) were isolated offline by filtering at 1–300 Hz. Spiking activity was measured by filtering the raw data at 500–3000 Hz, but because the quality of these high frequency data was variable over time and from animal to animal, likely because of changes in electrode impedance (that fortunately did not affect recorded LFPs), these data were not analyzed further. In the first recording session, approximate best frequency (BF) of the recording site was determined by presenting pure tone stimuli (50 ms duration, 5 ms cosine windowed rise/fall times) at 11 frequencies logarithmically spaced from 4.2 to 64 kHz, at 20–80 dB SPL in 20 dB steps. The frequency at which the LFP was detectable at the lowest intensity presented was taken as the BF. On occasion additional frequencies and/or intensities were presented to resolve ambiguity.

Multiple recording sessions were obtained in each animal (range 1–6 sessions, median = 3). In most animals (9/10) a single isoflurane concentration (subhypnotic = 0.4%, just-hypnotic = 0.8–0.9%, or reliably hypnotic = 1.6%) was selected for every experimental day, and data was obtained at baseline, drug and recovery conditions. The just-hypnotic concentration was selected as that causing loss of righting reflex (LORR) in that animal on that particular day. Baseline recordings were obtained for approximately 60 min, after which isoflurane was applied in room air. After reaching the desired concentration, 15 min were allowed for the animal to equilibrate and the drug applied for an additional 15 min to obtain the responses in the drug condition. Finally, the isoflurane was turned off and responses recorded continuously for 60 min at 0% isoflurane. In one animal, we recorded only at the just-hypnotic dose, in another only at the just- and reliably hypnotic doses, and in one animal, the isoflurane concentration was increased in a step wise manner, allowing recording with multiple isoflurane doses in one recording session.

In these recording sessions, stimuli consisted of pure tones, LED flashes, and paired LED-tone stimuli (11 stimuli in all for each recording session, randomly interleaved). Five different tones (50 ms duration) were chosen for each session: three at 40 dB SPL at BF, 1/2BF, and 2xBF, and an additional two at 20 and 60 dB SPL at BF. The five tones were presented alone and in combination with 1 ms, 0.37 cd-s/m^2^ LED flashes, with LED flashes preceding the tones at a stimulus onset asynchrony chosen to maximally align the visual and auditory responses, typically 65 ms. The eleventh stimulus in the set was the LED flash alone. LEDs were mounted on the head and positioned to be a constant 1 cm from the animal's eyes during the recording sessions. We note that the LED flash did not elicit any observable startle reflex in the animals. Animals were monitored via infrared video camera to ensure that their eyes remained open throughout the experiment, including when unconscious due to isoflurane. Responses to unilateral visual stimuli presented to the ipsilateral and contralateral eye were recorded, but as expected based on the known anatomy of the visual system in rats, ipsilateral stimuli were ineffective and not analyzed further.

#### Histological processing

**Figure 1:**
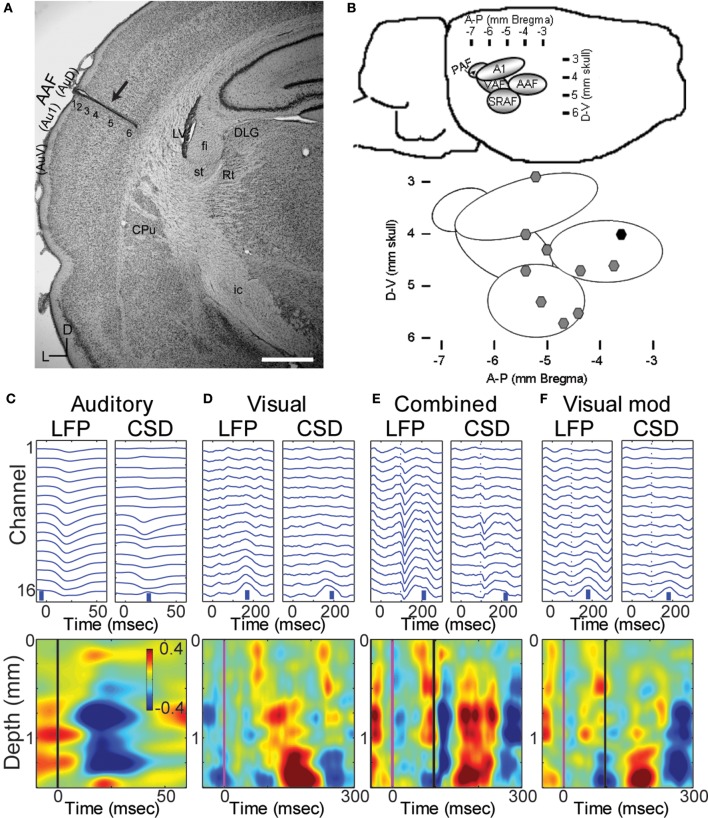
**Multichannel recordings of sensory responses from auditory cortex *in vivo*. (A)** Photomicrograph of a section through auditory cortex of a chronically implanted rat. The electrode trace can be clearly seen in auditory cortex (*arrow*). Scale bar in *lower right* is 1 mm. **(B)** Location of implanted probes: a map with coordinates of the auditory areas on the rat's brain (*top*) and the location of the implanted electrodes used in the manuscript on the map (*black symbol*: probe in **A**). **(C–F)** LFPs and derived CSD traces (*top row*) and CSD contour plots (*bottom row*) in response to a 50 ms tone burst at best frequency (13.4 kHz, 40 dB SPL; **C;** tone onset at time zero), a 1 ms LED flash (**D;** flash onset at time 0), combined stimulation **(E)** of the LED flash (at time 0) and best-frequency tone (at 96 ms), and the calculated visual modulation of the auditory response (**F;** difference between the combined and auditory responses). In CSD contour plots, sinks are indicated by blue and cool colors. Units for color bars are μA/mm^3^ throughout the manuscript. Scale bars (*vertical blue bars*) in top row of (**C–F**): 0.1 mV and 1 μA/mm^3^. Vertical lines in bottom row of **(C)** mark stimulus onsets: *black*, auditory; *magenta*, visual. Most anatomical terminology adopted from Polley et al. (2007), except “Au1,” “AuD,” and “AuV,” from Paxinos and Watson (2007). Cortical parcellation scheme in **(B)** adapted from Polley et al. (2007). Abbreviations: Au1, primary auditory cortex; AuV, secondary (ventral) auditory cortex; AuD, secondary (dorsal) auditory cortex; CPu, caudate putamen; LV, lateral ventricle; st, stria terminalis; fi, fimbria (hippocampus); Rt, reticular thalamic nucleus; ic, internal capsule; DLG, dorsal lateral geniculate nucleus; AAF, anterior auditory field; PAF, posterior auditory field; VAF, ventral auditory field; SRAF, suprarhinal auditory field.

Brain tissue was preserved histologically by means of previously described methods (Smith et al., 2010, 2012; Banks et al., 2011) to determine electrode track locations upon completion of *in vivo* recording experiments. In brief, rats were deeply anesthetized with sodium pentobarbital (90 mg/kg i.p.) and perfused intracardially with phosphate-buffered saline followed by 300–500 ml of an aldehyde fixative solution in sodium phosphate buffer. Coronal tissue sections 60 μm thick were cut from the fixed brain, mounted serially on slides, stained with Cresyl Violet, and coverslipped. Electrode entry, tracks, and tip position in the brain were determined by examination of serial sections using light microscopy camera lucida techniques. Locations in the brain were identified initially using the terminology and atlas of Paxinos and Watson (2007). Refer to Smith et al. (2012) for a full description of cytological features used to aid in identification of auditory cortical areas. Electrode sites were then mapped to corresponding functionally-defined auditory areas (Figure 1B) described in Polley et al. (2007) based on the dorsal-ventral and rostral-caudal position of the site of electrode entry. Digitized light level photomicrographs were acquired with a Spot camera (Diagnostic Instruments, Sterling Heights, MI) mounted on a Nikon Eclipse E600 microscope and prepared using Adobe Photoshop (San Jose, CA).

### Brain slice experiments

All reagents not specified below were obtained from Sigma-Aldrich (St. Louis, MO).

#### Brain slice preparation

Male B6CBAF1/J mice (*n* = 17 animals; median age = p38, range = p28–p98) were decapitated under isoflurane anesthesia, and the brains were extracted and immersed in modified artificial CSF [mACSF; composed of (in mM) 111 NaCl, 35 NaHCO_3_, 20 HEPES, 1.8 KCl, 1.05 CaCl_2_, 2.8 MgSO_4_, 1.2 KH_2_PO_4_, and 10 glucose] at 0–4°C. HEPES was included to improve slice health and prevent edema (Macgregor et al., 2001). Two types of slices were used. Auditory TC brain slices (450 μm; *n* = 10) were prepared from the right hemisphere as previously described (Cruikshank et al., 2002; Verbny et al., 2006). To record responses in auditory cortex to stimulation in extrastriate visual cortex, we also prepared coronal slices (450 μm; *n* = 7) from both hemispheres using standard techniques, as described (Banks et al., 2011). In these latter slices, we observed that the most consistent responses to V2 stimulation were observed in slices cut ~15° off the coronal plane, with the dorsal edge of the slice caudal to the ventral edge. Slices were maintained in mACSF saturated with 95% O_2_/5% CO_2_ at 24°C for >1 h before transfer to the recording chamber, which was perfused at 4–6 ml/min with ACSF [composed of (in mM) 111 NaCl, 35 NaHCO_3_, 20 HEPES, 1.8 KCl, 2.1 CaCl_2_, 1.4 MgSO_4_, 1.2 KH_2_PO_4_, and 10 glucose] at 30–34°C. In TC slices, primary auditory cortex was identified based on its position relative to the hippocampus and strong responses to stimulation of thalamic afferents, as in previous studies (Verbny et al., 2006). In coronal slices, primary auditory and extrastriate visual cortex were identified based on their position relative to the rhinal sulcus, midline and hippocampus, as described (Banks et al., 2011). Cortical layers were identified by differences in cell density and based on distance from the pia, as in previous studies (Verbny et al., 2006; Banks et al., 2011). We further used the tissue appearance under bright field illumination to identify the approximate borders between cortical layers. Layers 4 had a relatively dark appearance compared to the light colored bands of layers 3, 5, and 6.

#### Electrophysiological recordings

LFPs were recorded using silicon multi-electrode arrays consisting of 16 shanks (15 μm thick, 100 μm spacing) each with one iridium recording site (A16; Neuronexus Technologies). Data were amplified (HS-16, Lynx8; Neuralynx, Bozeman, MT), low-pass filtered (10 kHz), digitized (20 kHz; DigiData 1322A; Molecular Devices, City, State), and recorded using pClamp version 9.2 (Molecular Devices). Afferents were activated using pairs of tungsten electrodes (0.1 MΩ, 75 μm diameter; FHC Inc., Bowdoin, ME) cemented together at tip separations of ~50–200 μm. In coronal slices, stimuli were applied to layer 5 in V2 (see **Figure 6B**), as described (Banks et al., 2011). In TC slices, stimuli were applied to the superior thalamic radiation, just rostral to the hippocampus (Verbny et al., 2006), and to layer 1, ~1 mm rostral to the recording site (see **Figure 6A**). Stimuli (100 μs, 50–200 μA) were applied using constant current stimulus isolation units (A365, World Precision Instruments, Sarasota, FL) and consisted of either single pulses or brief trains (4 pulses, 40 Hz). Throughout, we refer to the L1 and V2/L5 stimuli as cortico-cortical (CC) stimuli, but we note that the L1 stimulus could also activate matrix TC afferents.

#### Anesthetic application

Isoflurane (0.5%, 1%, and 2%; Novaplus; Abbott Labs, N. Chicago, IL) was bath applied to slices from a 500 ml Teflon gas sampling bags (Fisher Scientific International Inc., Hampton, NH; cat. No. 10-923-5). Isoflurane was prepared as an aqueous solution either from a saturated 95% O_2_–5% CO_2_ gas diluted to final concentration in 50% gas (95% O_2_–5% CO_2_) and 50% ACSF in the sampling bags on the day of the experiment, or by bubbling 95% O_2_–5% CO_2_ into ACSF via Isoflurane vaporizer and measuring the gas concentration at the fluid surface with an anesthetic gas monitor (Poet II Anesthesia Monitor, Criticare Systems Inc., Waukesha, WI). Final Isoflurane concentrations in the bags were verified by either gas chromatography measurements (Gow-Mac Series 580 FID Isothermal Gas Chromatograph, Gow-Mac Instrument Co., Lehigh Valley, PA) of samples from each bag, or by sampling the gas concentration in the gas phase of the bag with an anesthetic gas monitor (Poet II Anesthesia Monitor, Criticare Systems Inc., Waukesha, WI) after 15 min of equilibration during shaking of the solution (“The belly dancer,” Stovall Life Sciences, Stovall, NC). Final anesthetic concentrations and electrophysiological results using the two methods were indistinguishable and were pooled in all analyses. Concentration measurements were used as covariates in statistical analysis of the data in **Figures 8C,D**.

### Data analysis

#### Neuronal data analysis

LFP responses to the different stimuli presented (both *in vivo* and in slices) were averaged for each channel triggered on the stimuli. Separate averaging was performed for each recording condition (control, isoflurane and recovery) in each recording session. To obtain steady state effects of isoflurane, LFPs were averaged only following 15 min or more of the drug application *in vivo*. For animals in which we performed more than one recording session in a certain isoflurane concentration we averaged the LFP responses over the corresponding sessions.

Current source density (CSD) (Mitzdorf, 1985) of the averaged LFP responses were estimated using either the spline or delta inverse CSD method (Pettersen et al., 2006). Briefly, trans-membrane currents flowing in neurons establish a time varying distribution of net current sources and sinks that constitutes a CSD distribution. These sources and sinks give rise to currents flowing in the extracellular space that are recorded as LFPs. Thus, the underlying CSD distribution can be calculated from LFP measurements, specifically by taking the second spatial derivative of the LFP measurements. Due to the relatively homogeneous geometry of neocortical tissue, when a linear electrode array is oriented perpendicularly to the cortical surface and LFPs are sampled at a fine enough spatial scale (inter-electrode spacing = 100 μm), the CSD distribution can be estimated using the standard solution technique as:

$$I_{m}=\frac{k*[\varphi (z+\Delta z)-2\varphi (z)+\varphi (z-\Delta z)]}{\Delta z^{2}}$$

Where φ(*z*) is the LFP measurement at depth *z*, Δ*z* is the inter-electrode spacing, and *k* is a conductivity constant. Positive values of *I_m_* correspond to net current sources, i.e., outward flowing current, and negative values correspond to net current sinks, i.e., inward flowing currents.

In order to determine the effect of anesthesia on the descending pathways, we calculated the difference between the response to a combined auditory + visual stimuli and the response to pure auditory stimuli for all five auditory stimuli presented in each experiment, then averaged the resulting five visual modulation responses (Figure 1F) and refer to it as the visual modulation response. This modulation response was nearly identical to the visual response alone, but because it could be derived from all five auditory stimuli presented, we recorded many more trials from which to calculate this response and it was often less noisy. Therefore, we used this visual modulation response for most analyses.

LFP responses *in vivo* were evaluated using the channel with the maximal peak response absolute value during the control period, and calculating the area under the peak response and above the significance line (average plus two standard deviation of the channel potential at rest). Response latency was calculated as the time from stimulus onset to 10% of the peak of the response.

Two types of measurements were derived from the CSD profile, one to measure the effect of isoflurane on the magnitude of the sink integral and one to measure the effect of isoflurane on the spatio-temporal response pattern. Response magnitude was calculated by first identifying the channel that displayed the maximal current sink within a pre-defined response window (*in vivo* auditory response: 10–100 ms post stimulus, search for maximum across all channels; *in vivo* visual modulation response: 20–300 ms after the visual stimulus, search for maximum across four deepest channels; slice TC and CC responses: 2–22 ms after the first stimulus in the 4 × 40 Hz train, search for maximum across all channels). Once the channel containing the peak CSD sink was identified, the CSD signal on this channel and the two channels immediately adjacent were thresholded (mean − 2 *SD*, computed over the pre-stimulus period) and integrated. To evaluate the effect of isoflurane on the spatio-temporal response pattern, the two-dimensional correlation coefficient of the CSD profile within the response window as defined above was calculated between the control (pre-drug) and drug and recovery conditions.

#### Statistical analysis

Statistical analyses were performed in SPSS (v22, IBM). To focus on the effects of isoflurane *per se* and not differences in response magnitude for different stimuli, the data were first normalized by dividing all the data across all conditions for each stimulus to the mean of the control data (across experiments) for that stimulus. For the *in vivo* LFP data of **Figure 4B** and the *in vivo* CSD sink data of **Figure 5C**, repeated measures analysis of variance (“GLM > Repeated Measures” in SPSS) was used to determine whether isoflurane had a differential effect on the auditory vs. visual modulation responses, with condition (control, drug, recovery) as the within-subjects factor and stimulus (auditory, visual) as the between-subjects factor. The reported ANOVA parameters (F statistic, *p*-value, and effect size) are on the condition ^*^ stimulus interaction term. The effect size presented is partial η^2^, which ranges from 0 (i.e., no effect) to 1 and corresponds to the fraction of variance accounted for by this interaction after controlling for other sources of variability. Because for most animals the data were collected separately for each concentration of isoflurane, the analysis was run independently for each drug concentration and the significance level was corrected for multiple comparisons to 0.017 (=0.05/3). For the cross correlation analysis of *in vivo* responses (**Figure 5D**), the control condition always has a value of 1, and thus we used paired Student's *t*-tests at each concentration, with the significance level corrected as above. For the brain slice data of **Figure 8**, experiments were typically conducted with five conditions (control, 0.5%, 1%, and 2% isoflurane, and recovery), but in 6 of 25 slices there were missing data points, either experiments in which only 1 or 2 of the isoflurane concentrations were tested (*n* = 4 slices) or experiments that terminated before recovery data could be obtained (*n* = 2 slices). These missing data points required a slightly different approach in SPSS, a linear mixed model analysis, to investigate the differential effect of isoflurane on TC vs. L1 and V2/L5 responses. Two approaches were used. For both approaches, because the results for the two CC stimuli were indistinguishable, these data were pooled and compared to TC responses. In the first, we analyzed the data using drug condition as a five level factor (**Figures 8A,B**), which allowed explicit comparisons at each isoflurane concentration by comparing the interaction term parameter estimates, i.e., the slopes on the fitted regression lines. As for the *in vivo* data, stimulus (TC, CC) was the between-subjects factor and the reported F statistic is on the condition ^*^ stimulus interaction term. In the second approach, we treated measured isoflurane concentration as a covariate (**Figures 8C,D**). Measured concentrations at nominal 0.5%, 1%, and 2% isoflurane for TC response data were 0.48 ± 0.035%, 1.0 ± 0.064%, and 2.0 ± 0.14% and for CC response data were 0.48 ± 0.068%, 0.99 ± 0.14%, and 2.0 ± 0.31%.

The LFP data of **Figure 4B**, and the sink integral data of **Figures 5C, 8A** deviated significantly from normality (Kolmogorov–Smirnoff test, *p* < 0.05); log-transformation alleviated this problem, and the analysis was run on these log-transformed data. Zeros in the *in vivo* data (corresponding to cases where no significant sink was detected) were replaced by 10^−3^ for the statistical analysis only. The specific choice of this replacement value had no qualitative effect on the results of the analysis. No log transformation was necessary for the cross correlation data of **Figures 5D**, **8B**. Results are presented as mean ± *SD* for data that could be described by a normal distribution, and as median [1st quartile, 3rd quartile] for data that deviated significantly from normality.

## Results

### *In vivo* electrophysiology

The data presented here were obtained from 10 animals in which probes were localized to a primary auditory field (Figures 1A,B), probes penetrated at least to layer 5 (Figure 1A) and responses to both auditory and visual stimuli could be identified in the LFP (Figure 1C). Angles of entry were close to 0 degrees (i.e., normal to the surface) in both the dorsal-ventral and anterior-posterior dimension (mean ± *SD*: 3.5 ± 6.9° D-V, 2.5 ± 2.6° A-P).

#### Responses to auditory, visual, and bimodal stimuli

Pure tones at BF elicited large and well-timed LFP responses, which corresponded to a stereotypical CSD response profile (Figure 1C). Shortest latency of significant LFP responses (12.3 ± 2.2 ms) were observed in the middle layers (0.9 ± 0.3 mm), as expected for responses mediated by core TC afferents (Scheel, 1988; Roger and Arnault, 1989; Romanski and Ledoux, 1993; Winer et al., 1999; Polley et al., 2007; Storace et al., 2010; Smith et al., 2012). Brief, early sinks were also observed in the deepest layers, consistent with direct projections to layer 6 from the auditory thalamus (Huang and Winer, 2000; Smith et al., 2012), and consistent with previous reports (Szymanski et al., 2009; Constantinople and Bruno, 2013). Subsequent to these presumably monosynaptic TC sinks, activity spread to supra- and infragranular layers (Figure 1C).

**Figure 2:**
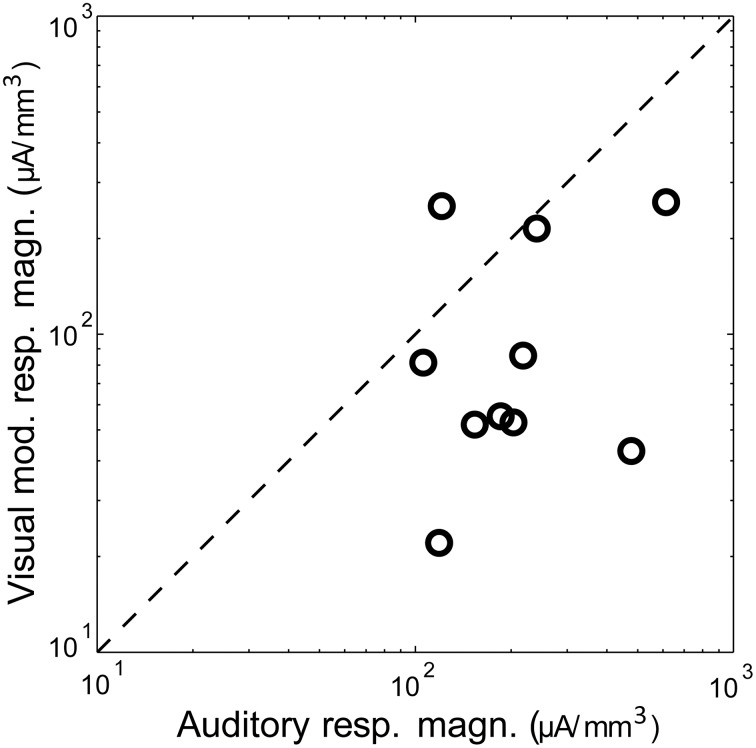
**Magnitude of auditory vs. visual modulation response**. Shown is the magnitude (area under the significant peak, i.e., >mean + 2 *SD* of baseline) of the visual modulation response as a function of the auditory response in the same animal, recorded under control conditions. The diagonal is the identity line.

Visual stimuli elicited long latency (~50 ms), long lasting (~250 ms) responses in primary auditory cortex, consistent with previous reports of multimodal responses in primary sensory cortex (Besle et al., 2009; Bizley and King, 2009; Doehrmann et al., 2010) (Figure 1D). Voltage amplitudes of visual responses were typically smaller than those of BF tones, but in some animals were comparable in size (Figure 2). CSD profiles typically had an alternating sink-source-sink pattern that was maximal in infragranular layers (Figure 1D). Visual stimuli presented before or simultaneous with auditory stimuli modulated auditory responses (Figure 1E). This modulation was mostly linear, and the visual modulation response, i.e., the difference between the paired and auditory alone responses (Figure 1F), was used for most subsequent analyses.

#### Effects of isoflurane on sensory responses *in vivo*

In order to determine isoflurane dose, we measured the minimal concentration required to achieve reliable LORR in 27 rats (including all the animals participating in this study). The LORR isoflurane dose was 0.86 ± 0.06%. We therefore used three isoflurane concentrations: sub-hypnotic (0.4%) in which the rats were active and responsive, just-hypnotic (0.8–0.9%) the dose in which LORR was obtained, and deep (surgical) anesthesia (1.6%).

**Figure 3:**
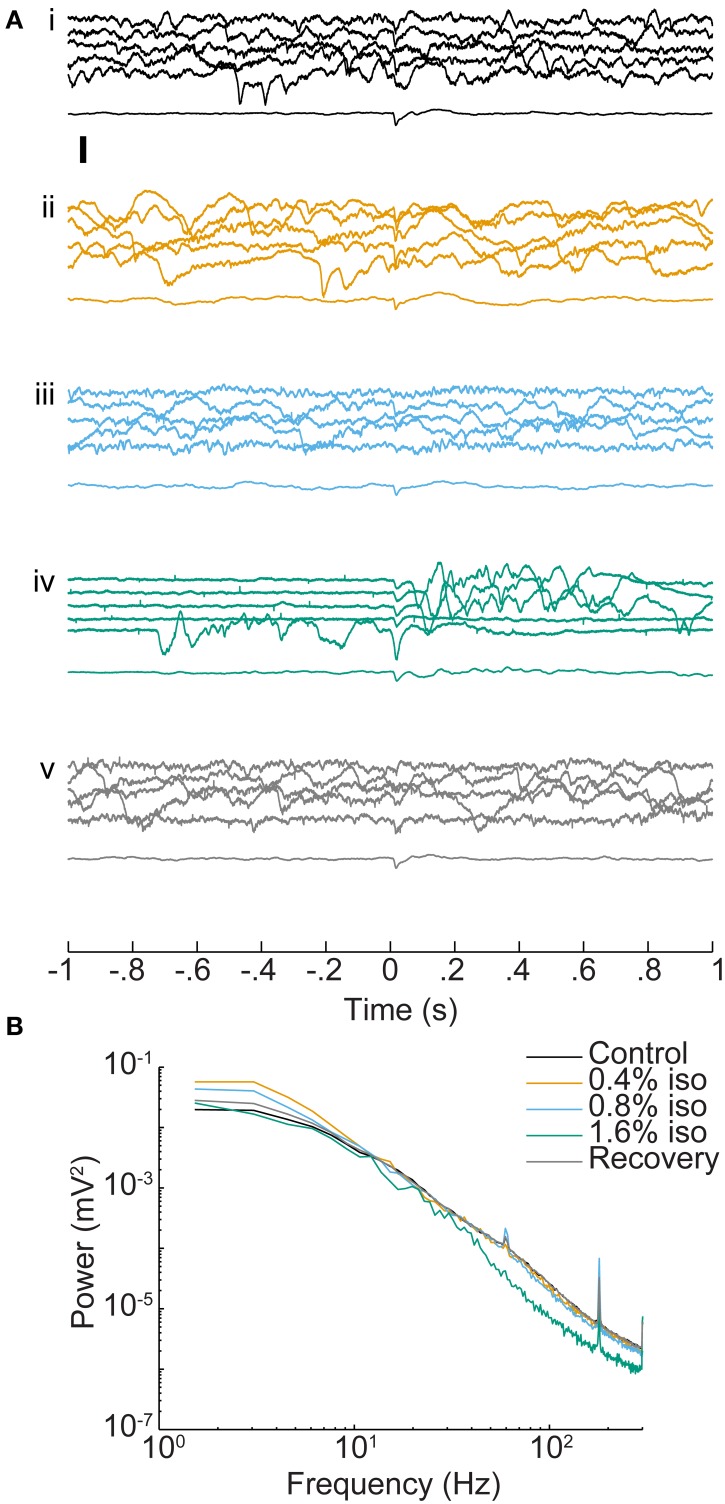
**Effect of isoflurane on spontaneous and sensory evoked responses in auditory cortex. (A)** LFPs recorded from one electrode (depth = 1 mm) during control conditions **(i)**, under increasing doses of isoflurane (**ii–iv**) and during recovery **(v)**. In each set, the top five traces are consecutive single trial LFPs and the bottom trace is the average of 20 responses. Stimulus onset is at *t* = 0 and was a BF tone at 60 dB SPL. Note the appearance of burst-suppression at 1.6% isoflurane and the occurrence of both spontaneous and stimulus-evoked bursts. Scale bar: 1 mV. **(B)** Mean power spectra of pre-stimulus activity derived from the animal in **(A)**. Spectra are the average single trial spectra of 220 trials under each drug condition, and control and recovery traces are averaged across three experiments.

Isoflurane had dose-dependent effects on ongoing activity in auditory cortex, but only modest effects on average evoked responses (Figure 3A). The most dramatic effect was burst-suppression (Hartikainen et al., 1995; Detsch et al., 2002), i.e., quiescence punctuated by spontaneous bursts, especially at 1.6% isoflurane (Figure 3Aiv). Spectral analysis of spontaneous activity showed enhancement of low frequency LFP components at lower doses of isoflurane and suppression of higher frequency components at 1.6% isoflurane (Figure 3B), as reported previously (Lukatch et al., 2005; Hudetz et al., 2011). Sensory stimuli also triggered burst responses that were indistinguishable from spontaneous bursts, as reported in visual cortex previously (Hudetz and Imas, 2007).

**Figure 4:**
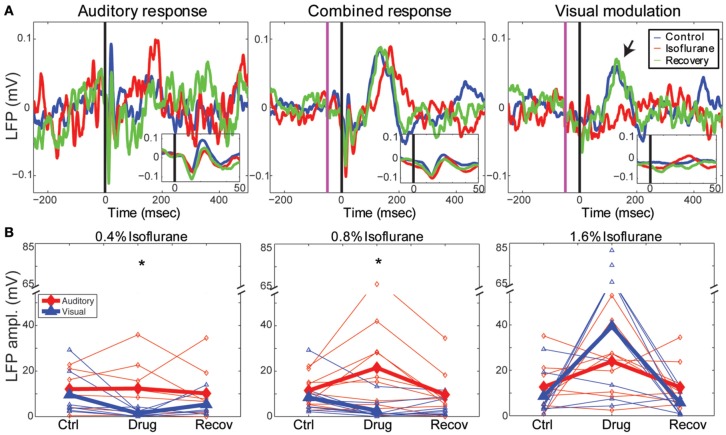
**Effects of isoflurane on auditory and visual responses. (A)** Single channel LFP responses to a 50 ms best-frequency tone burst (17.4 kHz, 60 dB SPL) at *t* = 0 *(left)*, combined stimulation, i.e., 1 ms LED flash preceding the same tone burst by 65 ms *(middle)* and the calculated visual modulation response (difference between the combined response and the auditory; *right)*. *Blue*: control; *red*: 0.8% isoflurane; *green*: recovery. *Insets* show early response components on expanded time scale. Note visual modulation response component between *t* = 100 and 200 ms (*arrow*) that is suppressed by isoflurane. **(B)** Summary of drug effects across animals. Plotted are LFP magnitude (area under the peak LFP response) of auditory (blue) and modulation (red) responses under 0.4% (*left*), 0.8–0.9% (*middle*) and 1.6% isoflurane (*right*). *Open symbols*: individual animals; *closed symbols*: mean across animals. ^*^p < 0.05, repeated measures ANOVA.

In Figure 4A, we show an example of the LFP responses to the auditory stimulus (left), combined stimulus (middle) and calculated visual modulation (right) under the different drug conditions recorded on one channel (1.4 mm depth). It can be seen that auditory LFP response are minimally affected by isoflurane whereas the visual modulation response is decreased. In order to quantify this effect we computed the response magnitude, defined as the area under the maximal peak of the LFP response for each animal (see Methods). As can be seen in the figure, the auditory response was mostly unchanged by sub-hypnotic dose, and increased by larger doses. The visual modulation magnitude, on the other hand, decreased under sub-hypnotic and just-hypnotic doses. The effect on the magnitude of the auditory and the visual modulation responses (calculated separately at each concentration; Figure 4B) was significantly different at the sub-hypnotic and just-hypnotic isoflurane concentrations [0.4% iso: *F*_(2, 28)_ = 7.32, *p* = 0.0105, partial η^2^ = 0.343; 0.8% iso: *F*_(2, 36)_ = 8.71, *p* = 0.0114, partial η^2^ = 0.326; 1.6% iso: *F*_(2, 32)_ = 1.63, *p* = 0.214, partial η^2^ = 0.0926; repeated measures ANOVA; see Methods]. *Post-hoc* tests for the sub-hypnotic and just-hypnotic cases showed significant differences between auditory and visual modulation responses for the drug condition but not the control or recovery conditions (*p* = 0.0185 and *p* = 0.00278 for 0.4% and 0.8–0.9%, respectively). The paradoxical increase at the highest concentration of isoflurane was due to late bursting activity elicited by the visual stimulus, as previously reported in visual cortex (Imas et al., 2004, 2005b). At this concentration, burst suppression was observed in the ongoing cortical activity, and sensory stimuli of both modalities often triggered burst responses. However, as visual stimuli triggered bursts more frequently than auditory stimuli, the response magnitude of the visual response increased to a greater extent in this condition.

**Figure 5:**
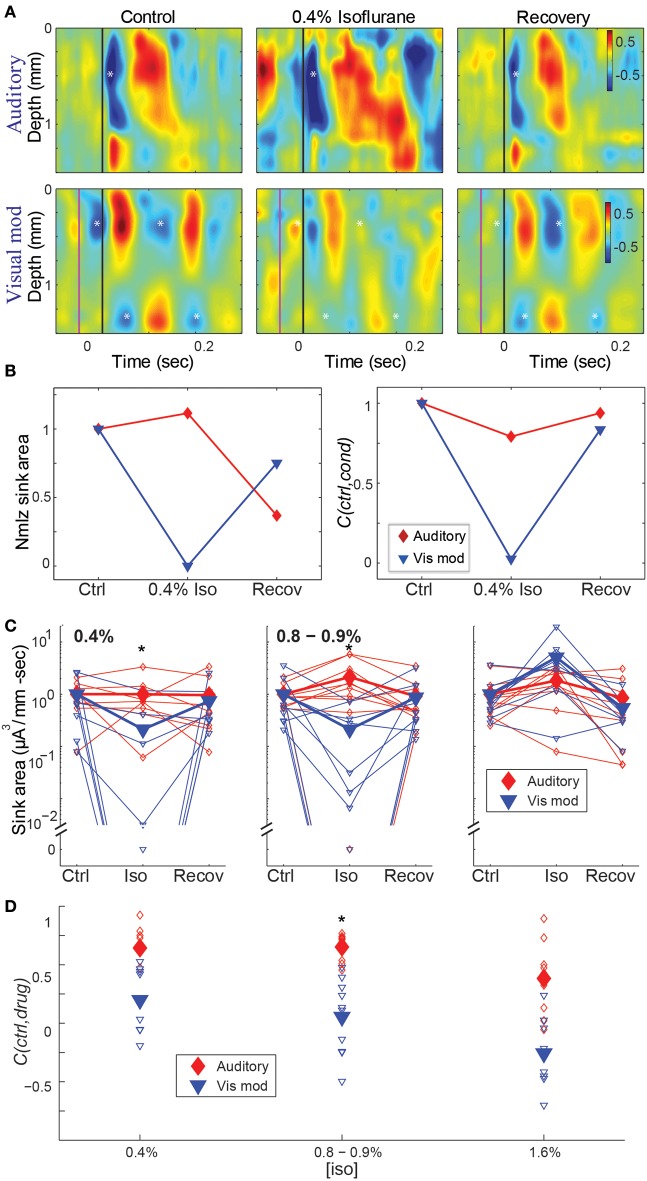
**Effect of isoflurane on spatiotemporal activity patterns in auditory cortex. (A)** CSD profiles of responses recorded in one animal to an auditory stimulus (50 ms tone, 17.4 kHz, 40 dB SPL) alone (*top*) and visual modulation of the auditory response (*bottom*) recorded in room air (*left*), 0.4% isoflurane (*center*) and recovery (*right*). Note early auditory response component (*asterisk*) that is relatively unaffected by isoflurane, and alternating supra- and infragranular sinks during visual modulation response (*asterisks*) that are suppressed by isoflurane. In *center* and *right* panels, asterisks are plotted at the same relative positions as in *left* panel. **(B)** Normalized response magnitude (*left*; normalized to the average of the control values) and correlation coefficient between control and drug and recovery responses (*C*(*ctrl,cond*)) for the data in **(A)** (*red*: auditory; *blue*: visual modulation). **(C–D)** Summary across animals. Magnitude **(C)** and correlation coefficient **(D)** of the auditory (*red*) and visual modulation (*blue*) responses under different isoflurane concentrations. *Open symbols*: single animals; *filled symbols*: average across animals. ^*^p < 0.05, repeated measures ANOVA, in **(C)** and *p* < 0.017, paired Student's *t*-test, in **(D)**.

The effects of isoflurane on CSD responses in auditory cortex were modality specific, with greater suppression of visual modulation responses at sub-hypnotic and just-hypnotic isoflurane concentrations (Figure 5). We used two measures to quantify the effect of isoflurane on CSD responses. First, to measure the effect of isoflurane on the magnitude of the response, we calculated the integral of the major current sink for each stimulus under control conditions (see Methods), and compared this measurement to the integral of the current sink at the same spatial location and time window under isoflurane and upon recovery. Second, to measure the effect of isoflurane on the spatio-temporal pattern of the response, we computed the averaged CSD responses under control conditions, and calculated the 2-dimensional correlation coefficient between this control response and the drug response (*C*(*ctrl,drug*)), within standardized response windows.

Using both measures, isoflurane had a greater effect on visual modulation compared to auditory responses (Figures 5C,D). Auditory responses were largely unaffected at 0.4% isoflurane (median ratio of drug to control [1st quartile, 3rd quartile]: 1.13 [0.317, 1.54]), and enhanced at 0.8% and 1.6% (1.84 [0.759, 3.53] and 1.91 [0.965, 8.66], respectively), whereas visual modulation responses were suppressed at 0.4% and 0.8% and enhanced at 1.6% (0.0245 [0.00, 0.622], 0.010 [0.00, 0.459] and 6.09 [3.41, 12.2], respectively). There was a significant difference in the effect of isoflurane on the sink area of visual modulation vs. auditory responses at 0.4% and 0.8 – 0.9%, but not at 1.6% isoflurane [Figure 5C; 0.4% iso: *F*_(2, 28)_ = 6.22, *p* = 0.00583, partial η^2^ = 0.308; 0.8–0.9% iso: *F*_(2, 36)_ = 7.96, *p* = 0.00553, partial η^2^ = 0.307; 1.6% iso: *F*_(2, 32)_ = 1.04, *p* = 0.355, partial η^2^ = 0.0610; repeated measures ANOVA]. *Post-hoc* tests for the sub-hypnotic and just-hypnotic cases showed significant differences between the auditory and visual modulation responses for the drug condition but not the control or recovery conditions (*p* = 0.00318 and *p* = 0.00912 for 0.4% and 0.8–0.9%, respectively). Differential effects of isoflurane on auditory vs. visual modulation responses were also observed for the correlation coefficient, though the effect reached statistical significance only at the just-hypnotic concentration [Figure 5D; mean ± *SD C(ctrl,drug)* for 0.4% iso: aud, 0.64 ± 0.22; vis mod, 0.20 ± 0.29, *p* = 0.0500; for 0.8–0.9% iso: aud, 0.65 ± 0.11; vis, 0.054 ± 0.32, *p* = 0.00122; for 1.6% iso: aud, 0.38 ± 0.31; vis mod, −0.26 ± 0.29, *p* = 0.0192; paired Student's *t*-tests]. As for the LFP data of Figure 4B, the paradoxical increase in sink magnitude at 1.6% isoflurane was due to long-latency bursting elicited by visual and auditory stimuli during burst suppression.

### Brain slice electrophysiology

**Figure 6:**
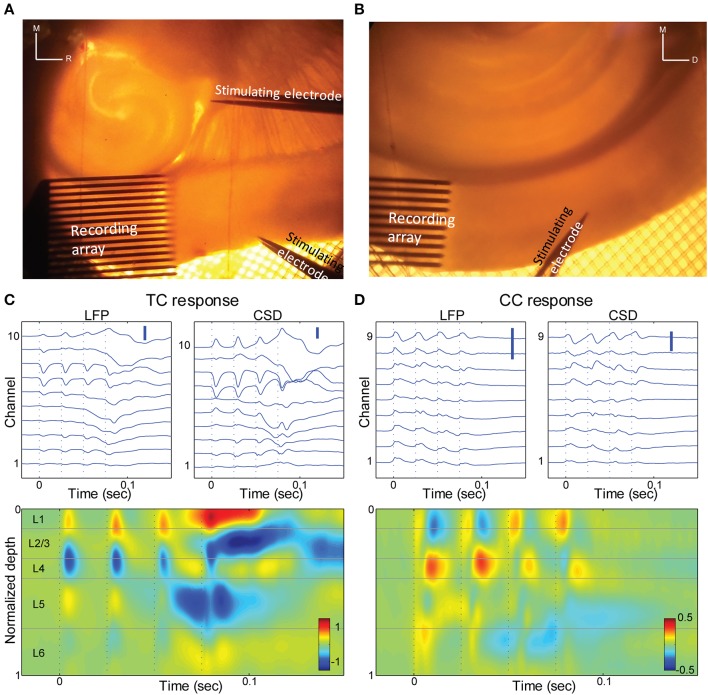
**Thalamo-cortical and cortico-cortical responses in brain slices. (A)** Photomicrograph of a TC slice. Recording array is in auditory cortex, and stimulating electrodes are in the TC afferent bundle and in layer 1 of a proximal cortical region. For scale reference, inter-electrode spacing in the recording array is 100 μm. **(B)** Photomicrograph of a coronal slice. Recording array is in auditory cortex, and stimulating electrodes in upper layer 5 of area V2. **(C)** LFP responses at different depths (*top left*) and derived CSD (*top right; bottom*) in response to a train of 4 × 40 Hz stimuli of the TC fibers. Same slice as in **(A)**. Only electrodes that were in the cortex are shown and were used for further calculations. Scale bars 0.1 mV and 1 μA/mm^3^. **(D)** Similar to **(C)**, but for stimulation of V2 in layer 5. Same slice as in **(B)** Scale bars 0.1 mV and 1 μA/mm^3^.

We have shown that isoflurane suppresses visual modulation of auditory responses recorded in primary auditory cortex *in vivo* to a greater extent than auditory responses. This modality-specific effect could be due to selective and local effects on synapses in auditory cortex carrying visual (from higher-order cortical areas and non-specific thalamus) vs. auditory (specific thalamic) information. Alternatively, isoflurane could have a greater effect on the sources of visual vs. auditory input to auditory cortex, with these effects reflected indirectly in our recordings. To examine whether isoflurane can produce this effect at the level of the auditory cortex independently of the effects on upstream areas, we investigated the effects of isoflurane on TC and CC responses in auditory cortical brain slices (Figure 6).

#### CSD responses to TC, L1, and V2/L5 stimulation

**Figure 7:**
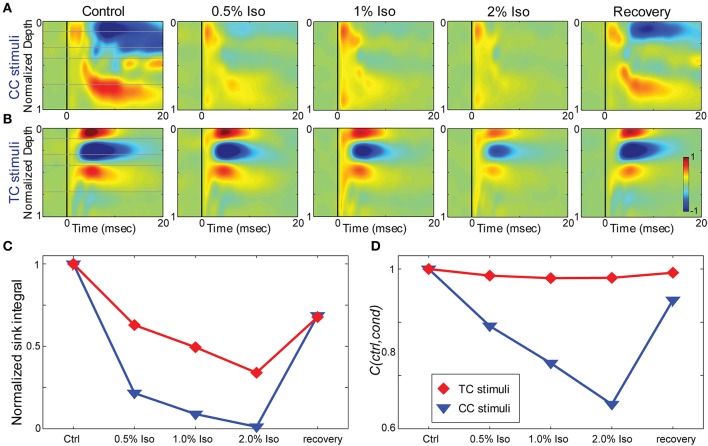
**Effects of isoflurane on synaptic responses in brain slices. (A,B)** CSD response to a L1 stimulus **(A)** and TC stimulus **(B)** in the same TC slice under increasing isoflurane doses (left to right: control, 0.5%, 1%, 2% and recovery). **(C)** Magnitude of the response (area under the maximal sink in response to the first stimulus in the train, normalized to the average of the control and recovery responses) for the data in **(A)** (*blue*) and **(B)** (*red*). **(D)** Correlation coefficient between the control response and each drug condition for the data in **(A,B)**; color code as in **(C)**.

We measured extracellular LFP responses in brain slices of auditory cortex to afferent stimulation using multi-channel electrode arrays in two different brain slice preparations. In TC slices (*n* = 10 slices) (Cruikshank et al., 2002; Verbny et al., 2006), we stimulated TC afferents and compared these responses to stimulation of CC afferents in L1 (Figures 6A,C, 7A,B). As we and others have shown previously (Cruikshank et al., 2002; Verbny et al., 2006), stimulation of the fiber pathway just rostral to the medial geniculate in auditory TC brain slices triggered short latency (2.3 ± 0.7 ms), presumably monosynaptic LFP responses that corresponded to current sinks in granular layers (Figure 6C). The spatial location of this short latency sink was similar to the initial current sink observed in middle layers *in vivo* in response to auditory stimuli.

To activate CC fibers, in 8 of these 10 TC slices we stimulated in layer 1 approximately 0.5–1 mm rostral to the recording site (Figure 6A). The spatial CSD profile of the responses to stimulation in layer 1 consisted of a short latency (5.9 ± 1.9 ms), presumably monosynaptic current sink. In most slices (7/8) this early sink was maximal in the supra-granular layers (4/8 in layer 1–2, 3/8 in layer 2–3; Figure 7A), consistent with the known anatomy of these fibers and with previous reports (Cauller and Connors, 1994). In one slice L1 stimulation elicited an early current sink in layer 5 (not shown). We note that the spatial profiles of these responses are distinct from the response to TC stimulation.

Responses were examined in coronal slices as well (*n* = 7 slices; Figure 6B), in which we were able to better isolate CC from the non-specific TC fibers that also travel in L1. In this preparation, slices were prepared that preserved the descending CC projection from extrastriate visual cortex (V2) to primary auditory cortex, as previously described (Banks et al., 2011). To activate V2, we stimulated in L5, where descending projection cells are concentrated (Felleman and Van Essen, 1991). The CSD response profile to V2 stimulation was again distinct from the TC response profile, with prominent short-latency (5.2 ± 2.0 ms), presumably monosynaptic current sinks observed either in infragranular layers (4/7 slices) or in the superficial layers (3/7 slices) (Figure 6D), as for L1 stimulation in TC slices. The latencies of the early TC evoked sinks were shorter than those of the different CC responses (*p* < 0.001, unpaired Student's *t*-test for both TC to L1 and TC to V2 comparisons), whereas there was no significant difference between the latencies of the two CC responses (*p* = 0.497, unpaired Student's *t*-test).

TC and CC afferent stimuli could also trigger longer latency polysynaptic activation of supra- and infragranular layers. This activity originated in layer 5 and often spread to more superficial layers (e.g., the late current sink in layer 5 at ~0.055 s in Figure 6C, *bottom*), and appeared as all-or-none network bursts; shorter latencies to polysynaptic activity were observed with higher stimulation strength or the presence of multiple stimuli in a train. This polysynaptic activity has been observed previously in auditory, visual, and somatosensory brain slice preparations (Metherate and Cruikshank, 1999; Sanchez-Vives and McCormick, 2000; Cruikshank et al., 2002; Maclean et al., 2005; Watson et al., 2008; Rigas and Castro-Alamancos, 2009), where it has been shown to be non-epileptiform in nature and represent an *in vitro* correlate of UP states that occur *in vivo* (Sanchez-Vives and McCormick, 2000; Shu et al., 2003; Cunningham et al., 2006; Rigas and Castro-Alamancos, 2007).

#### Effects of isoflurane on electrophysiological responses in brain slices

**Figure 8:**
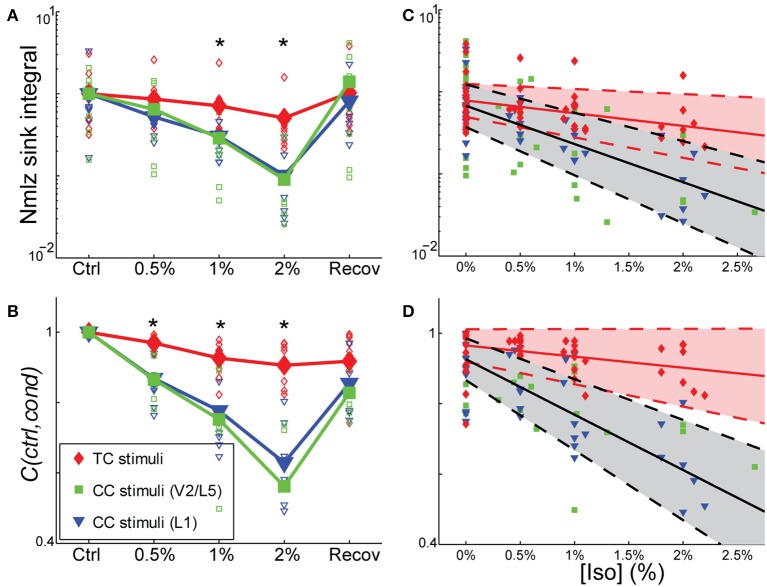
**Differential sensitivity of CC vs. TC pathways to isoflurane. (A,B)** Effect of isoflurane on sink integral **(A)** and 2D-cross correlation **(B)** of responses to TC (*red*), V2\L5 (*blue*), and layer 1 (*green*) stimuli under increasing isoflurane concentrations (left to right: control, 0.5%, 1%, 2% and recovery). Open marks represent single slices and filled marks represent the average. **(C,D)**. Linear mixed model fits to sink integral **(C)** and 2D-cross correlation **(D)** of responses to TC and CC stimuli plotted as function of actual measured isoflurane concentration. Shaded areas (*red*: TC; *gray*: CC) represent 95% confidence bounds for the model fits. Note the similarity in the effects of isoflurane on responses to the two types of CC stimuli (obtained in two different slice preparations), whereas the TC response is much less affected. ^*^p < 0.05, repeated measures ANOVA.

To determine whether the effects of isoflurane observed *in vivo* could be accounted for by local effects of the drug in auditory cortex, we applied isoflurane dissolved in ACSF to brain slices and measured the effects on CSD responses to stimulation of TC and CC (L1 and V2/L5) pathways. Three concentrations of isoflurane were applied (0.5%, 1%, and 2%), corresponding approximately to the three concentrations employed *in vivo* after taking into account loss of isoflurane gas in the recording chamber (see Methods). Examples of the effect of isoflurane on short latency synaptic responses can be seen in Figure 7, which we focus on for all further analysis. Polysynaptic activity driven by both TC and CC stimuli were suppressed by isoflurane (not shown). Consistent with our observations *in vivo*, bath application of isoflurane suppressed short latency L1 and V2/L5 responses in brain slices to a greater extent than TC responses. As for the *in vivo* data, we measured the change in the response strength using the sink integral and the response pattern using the 2-D correlation coefficient. Although isoflurane reduced the magnitude of both TC and CC sink integrals, it had a significantly greater effect on CC responses compared to TC responses at 1% and 2% isoflurane (Figures 8A,C). Because the effects of isoflurane on L1 and V2/L5 responses were indistinguishable, we pooled these data and compared to the effects on TC responses. We found that the sink integral was suppressed by isoflurane to a greater extent at each concentration tested (TC vs. CC median ratio of drug to control [1st quartile, 3rd quartile] at 0.5% iso: 0.836 [0.656, 1.06] vs. 0.616 [0.379, 0.784]; 1% iso: 0.737 [0.521, 0.856] vs. 0.379 [0.248, 0.506]; 2% iso: 0.356 [0.312, 0.649] vs. 0.088 [0.0400, 0.281]). Statistical analysis using a linear mixed model (see Methods) to compare the effects of isoflurane on TC vs. CC responses found a significant effect overall using drug condition as a categorical factor [*F*_(4, 82.4)_ = 9.07, *p* = 4.00e-6], with significant differences in the interaction terms at 1% and 2% isoflurane [*t*_(−2.88)_, *p* = 5.023e-3 and *t*_(−5.34)_, *p* = 8.17e-7, respectively]. Similar results were obtained when measured isoflurane concentration was included as a covariate [instead of drug condition as a factor; *F*_(1, 88.7)_ = 26.2, *p* = 2.00e-6; slopes and 95% confidence intervals for the stimulus ^*^ [iso] terms were −0.155 [−0.248, −0.062] for TC and −0.465 [−0.580, −0.345] for CC]. Converting back from logarithmic units, this tells us that the slope of the iso effect on the TC response was about −30% change/unit % isoflurane, compared to a slope of >−65% change/unit % isoflurane for the CC response, i.e., a suppressive effect that is more than twice as strong for CC vs. TC responses.

Similar effects on the response pattern were observed by measuring the response window correlation coefficient, but in this case significant differences between effects on TC and CC responses were observed at all three concentrations of isoflurane (Figures 8B,D; mean ± *SD C(ctrl,drug)* for TC vs. CC at 0.5% iso: 0.97 ± 0.020 vs. 0.87 ± 0.069; 1% iso: 0.93 ± 0.050 vs. 0.77 ± 0.13; 2% iso: 0.91 ± 0.061 vs. 0.60 ± 0.15). Statistical analysis showed a significant effect overall using drug condition as a categorical factor [*F*_(4, 83.2)_ = 19.2, *p* = 3.30e-11], and significant effects at all three concentrations of isoflurane [effects on interaction term for 0.5% iso: *t*_(−2.97)_, *p* = 3.94e-3; 1% iso: *t*_(−4.58)_, *p* = 1.6e-5; 2% iso: *t*_(−8.30)_, *p* = 1.56e-12]. Similar results were obtained when measured isoflurane concentration was included as a covariate [instead of drug condition as a factor; *F*_(1, 90.5)_ = 36.5, *p* = 3.38e-8; slopes and confidence intervals for the stimulus ^*^ [iso] terms were −0.0316 [−0.0637, 0.000545] for TC and −0.156 [−0.199, −0.115] for CC]. Thus, the slope of the iso effect on the TC response was about −7% change/unit % isoflurane, compared to a slope of >−30% change/unit % isoflurane for the CC response, i.e., an effect that is more than four times as strong for CC vs. TC responses.

**Figure 9:**
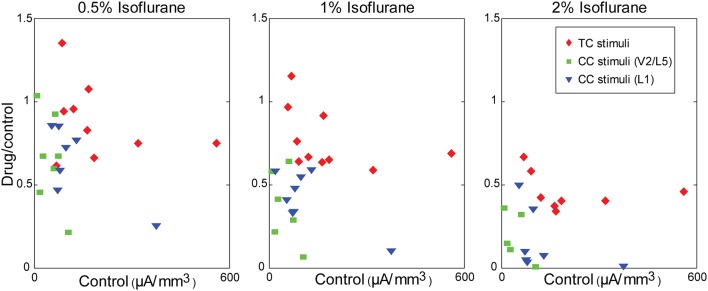
**Modulation of synaptic responses in slices by isoflurane is independent of control response magnitude**. Ratio of the response in drug to the response in control, plotted as a function of the control response magnitude, at different isoflurane concentrations (left to right: 0.5%, 1%, 2%). Note the dose-dependency of the isoflurane effect (i.e., compare across panels), but that small responses within any given panel are not more affected by isoflurane than large responses.

Although the magnitude of CC responses was on average smaller than TC responses under control conditions, the larger magnitude of the TC response did not play a role in the differential effect of isoflurane. This can be seen in plots of the ratio of the response under each drug condition to the control response as a function of the control response magnitude (Figure 9). Larger effects of isoflurane would manifest as scatter plots with positive slopes; by contrast, in all cases the data exhibited significant negative slopes (not shown), indicating that stronger responses had a slight tendency to be more suppressed by isoflurane.

**Figure 10:**
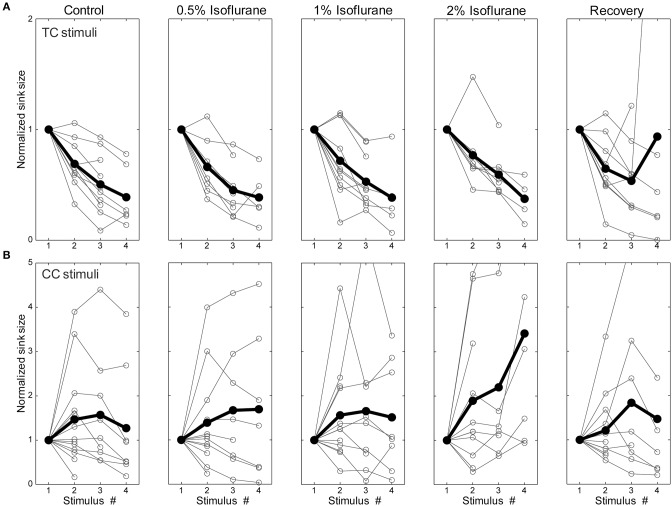
**Short-term plasticity was not changed by isoflurane**. Synaptic current sink magnitude during a 4 × 40 Hz train normalized to the response to the first stimulus in the train under different isoflurane concentrations (left to right: control, 0.5%, 1%, 2%, and recovery) for TC **(A)** and CC **(B)** stimuli. Horizontal axis is the stimulus number within the train. Note that although trains of 4 stimuli were presented in all experiments, only sinks that differed significantly from baseline (i.e., >mean + 2 *SD*) are plotted in the figure. It can be seen that TC stimulation always displayed short-term depression whereas CC stimulation could display either depression or facilitation. However, isoflurane did not affect this short-term plasticity.

The observation that isoflurane decreases the magnitude of synaptic current sinks in brain slices suggests a local action of isoflurane on the TC network. Previous studies have demonstrated that volatile anesthetics can suppress synaptic responses by acting presynaptically to reduce neurotransmitter release (Perouansky et al., 1995; Maciver et al., 1996; Kirson et al., 1998). One possible mechanism for the differential effects of isoflurane observed here is greater sensitivity of CC synaptic terminals in supra- and infragranular layers to such suppressive effects compared to TC synaptic terminals in granular layers. We investigated this issue by evaluating effects of isoflurane on short-term plasticity of responses to TC, L1, and V2/L5 stimulation by presenting trains of stimuli at 40 Hz. Under control conditions, TC pathways exhibited short-term depression, whereas L1 and V2/L5 exhibited a wide range of plasticity including both facilitation and depression (Figures 10A,B—left column). The short-term plasticity in the L1 and V2/L5 responses was indistinguishable, and to examine the effect of isoflurane these data were pooled together. Interestingly, we observed that the isoflurane effect on short-term plasticity was minimal in both TC and CC pathways. Statistical analysis of the ratio of the fourth to first response in the 4 × 40 Hz train indicated no effect of drug condition [TC: *F*_(4, 28)_ = 0.716, *p* = 0.428, partial η^2^ = 0.093; CC: *F*_(4, 36)_ = 1.368, *p* = 0.280, partial η^2^ = 0.132; repeated measures ANOVA run separately for TC and CC stimuli, within-subjects factor = condition (control, 0.5%, 1%, 2%, recovery)]. These data suggest that changes in release probabilities as manifested in short-term plasticity (Del Castillo and Katz, 1954; Zucker, 1989) do not play a major role in the effects of isoflurane.

## Discussion

### Pathway-specific actions of isoflurane

We have shown that LFP responses in auditory cortex to visual vs. auditory and CC vs. TC inputs are differentially modulated by the general anesthetic isoflurane. We distinguished bottom-up responses to acoustic stimuli *in vivo*, driven largely by ascending thalamic input from MGv (based on the laminar profile of current sinks), as well as responses *in vitro* to direct stimulation of the TC fiber pathway, and compared these to “top-down” responses, such as responses to visual stimuli *in vivo*, driven largely by descending CC and matrix thalamic input, as well as CC responses *in vitro* to stimulation in V2/L5 and L1. Isoflurane suppressed visual and CC responses to a greater extent than auditory and TC responses in auditory cortex, consistent with reports of differential effects on bottom-up vs. top-down connectivity derived from EEG and imaging data in humans (Imas et al., 2005a; Peltier et al., 2005; Alkire, 2008; Lee et al., 2009, 2013b; Ku et al., 2011; Liu et al., 2011; Schrouff et al., 2011; Boly et al., 2012). Our observations *in vitro* indicate that the effects of isoflurane observed *in vivo* can be accounted for largely by local, pathway-specific actions of isoflurane in auditory cortex. Results were remarkably consistent between V2/L5 and L1 stimulation in the two different slice preparations investigated (Figure 8), suggesting that the observed suppression of synaptic responses generalizes across multiple CC pathways. One difference between the results *in vivo* and *in vitro* that merits further investigation was that we observed a greater suppressive effect of isoflurane on TC responses in brain slices compared to auditory responses *in vivo* (see below). Although responses to CC stimuli were in general smaller than those to TC stimuli at comparable stimulation intensity, the differential effect of isoflurane cannot be explained simply by the magnitude of the synaptic response, i.e., greater suppression of smaller responses, as we observed no relationship between the effect of isoflurane and the magnitude of the response under control conditions (Figure 9).

### CSD responses under control conditions

Within a cortical column, the laminar segregation of synaptic terminals arising from afferent fibers as well as intra-columnar connections results in specific spatio-temporal patterns of activity that vary with the input fiber pathway engaged (Felleman and Van Essen, 1991). Responses to acoustic stimuli and to stimulation of core TC fibers elicited current sinks with largest amplitude and shortest latency in middle layers (Figure 1C), consistent with highest thalamic synaptic terminal density in granular layers 3 and 4 (Shi and Cassell, 1997; Huang and Winer, 2000; Smith et al., 2012). A secondary current sink of similar latency was sometimes observed in the deepest layers, consistent with the observed secondary projection of MGv to layer 6 (Smith et al., 2012). Similar CSD patterns have been observed previously in auditory cortex *in vivo* and *in vitro* (Cruikshank et al., 2002; Kaur et al., 2005; Lakatos et al., 2005; Szymanski et al., 2009).

In contrast to the CSD pattern elicited by TC stimulation, responses to CC stimuli were dominated by current sinks in either supra- (Figures 6B, 7A) or infragranular (not shown) layers. This sink/source pattern was complementary to that elicited by TC stimulation, analogous to the complementary nature of TC vs. CC afferent terminal density patterns (Smith et al., 2010, 2012; Banks et al., 2011), and is consistent with these stimuli engaging different afferent fiber pathways. Complimentary activation patterns have been observed in auditory cortex for acoustic and non-acoustic sensory stimulation previously (Lakatos et al., 2007, 2009).

Several papers have indicated that visual stimuli and eye movements can elicit responses on their own or alter responses to auditory stimuli in auditory cortical areas including primary auditory cortex (Fu et al., 2004; Besle et al., 2009; Bizley and King, 2009). The anatomical projections carrying visual information to auditory cortex terminate with highest density in supra- and infragranular layers (Miller and Vogt, 1984; Budinger et al., 2006; Bizley et al., 2007; Smith et al., 2010) in a classically top-down/modulatory pattern (Felleman and Van Essen, 1991; Shi and Cassell, 1997; Kimura et al., 2004). Cortical descending projections are postulated to carry expectation- and memory-based predictive information to be integrated with ascending sensory information (Bar, 2009; Bastos et al., 2012). Recent experimental observations (Covic and Sherman, 2011; De Pasquale and Sherman, 2011) and theoretical considerations (Bastos et al., 2012) suggest that these inputs may be both modulatory and driving. We found that the response to a combined auditory-visual stimuli was well-described by the linear sum of the two separate responses. Although some previous studies have shown super-additive effects of somatosensory and auditory inputs to auditory cortex (Ghazanfar et al., 2005; Lakatos et al., 2007), these effects were observed for near-threshold stimuli, whereas higher intensity stimuli, similar to what we employed in this study, were simply additive, as seen in our data (inverse effectiveness phenomenon).

Responses in slices were dominated by early, putatively monosynaptic current sinks that were of limited extent spatially and temporally, followed by late, burst responses reminiscent of UP state activity reported previously in cortical slices (Metherate and Cruikshank, 1999; Sanchez-Vives and McCormick, 2000; Cruikshank et al., 2002; Maclean et al., 2005; Watson et al., 2008; Rigas and Castro-Alamancos, 2009; Wester and Contreras, 2012). By contrast, CSD responses *in vivo* exhibited intermediate latency current sinks in supra- and infragranular layers and likely reflected polysynaptic sensory responses within the cortical column. Responses to visual sensory stimuli in auditory cortex *in vivo* and CC (V2/L5 and L1) stimulation in brain slices were less similar (compare Figures 1D and 6D), in that the latency and duration of visual responses were much longer than V2/L1 responses in slices, and sinks *in vitro* were confined to either the supra- or infragranular layer (usually the former), whereas *in vivo* we observed an alternating sink/source pattern that was most pronounced in the infragranular layers, and often alternated between infra- and supragranular layers (Figures 1D,F). These differences are not unexpected given the different stimulation paradigms utilized. Visual stimuli *in vivo* evoke long-latency responses in auditory cortex (Bizley et al., 2007; Kayser et al., 2008; Schroeder et al., 2008) due to slow transduction in the retina as well as the circuitous synaptic pathway carrying visual information to auditory cortex. The accumulated jitter in the latencies of visual thalamic and cortical cells will distribute temporally responses *in vivo*. By contrast, in our slice experiments, electrical stimulation of L1 or V2/L5 fiber pathways synchronously activated cells and fibers with monosynaptic, short distance projections to auditory cortex, resulting in shorter latency, less dispersed responses.

We used two different species for our recordings *in vivo* and brain slices. The reason for this was a practical one. The TC slice preparation has only been described for mice, and thus rats are unsuitable for the brain slice experiments. However, for *in vivo* recordings we wished to have both the LED mount and the 16-channel connector cemented into the animal's headcap, and the skulls of mice did not provide sufficient surface area to achieve this easily whereas rats' skulls did. Besides for the broad similarities apparent in cortical anatomy and physiology (Ehret, 1997; Stiebler et al., 1997; Kaur et al., 2005), we have shown that one of the afferent pathways central to the current study, the projection from V2 to A1, exhibits remarkable similarity between the two species (Smith et al., 2010; Banks et al., 2011). However, it is possible that subtle differences in connectivity and response properties in the two species contributed to some of the differences between our results *in vivo* and in brain slices.

### Modulation of responses by isoflurane

Several recent studies have emphasized the roles of brainstem and midbrain nuclei in acting as switches that control the arousal level and hypnotic effects of anesthetic agents (Devor and Zalkind, 2001; Nelson et al., 2002; Alkire et al., 2007; Langsjo et al., 2012; Solt et al., 2014), but there is overwhelming evidence that consciousness itself is a phenomenon of the cortico-thalamic network (Llinas et al., 1998; Crick and Koch, 2003; Tononi, 2004; Alkire et al., 2008b; Mashour, 2013). Anesthetic actions on nuclei involved in arousal and the sleep/wake cycle are likely to constitute on/off switches whose effects are mediated through actions in the cortico-thalamic network. The results presented here, as well as recent study demonstrating layer- and area-specific effects of anesthesia in cortex (Sellers et al., 2013), will aid in understanding mechanistically how actions in the cortico-thalamic network can mediate changes in consciousness.

Early studies on anesthetic modulation of cortical responses to auditory stimuli reported a reduction and slowing of field potentials recorded at the surface by several classes of anesthetic agents at surgical (i.e., higher than just-hypnotic) doses (Schwender et al., 1993b,c, 1994). These results indicated that the magnitude of mid-latency auditory evoked responses, which derive at least in part from activation of auditory cortex (Milner et al., 2014), could be used to predict the extent of verbal memory retention under anesthesia. Similar results have been obtained more recently in imaging studies in humans, which have shown general anesthetics suppress auditory cortical fMRI BOLD signals in response to musical or speech stimuli (Dueck et al., 2005; Kerssens et al., 2005; Plourde et al., 2006). Interestingly, decreased BOLD responses, and impaired memory, are observed even at sub-hypnotic doses, and thus may reflect effects on higher order cortical processing related to memory formation rather than stimulus identification *per se*. Consistent with this hypothesis, a more recent study reported that cortical responses to verbal stimuli are maintained in primary auditory cortex, but disrupted in higher order cortex, under deep propofol sedation (Liu et al., 2011).

In animal studies, in which primary sensory cortex can be targeted specifically and neuronal responses measured directly, it has long been known that sensory-evoked responses are maintained under anesthesia (Mountcastle et al., 1957; Hubel and Wiesel, 1959; Merzenich et al., 1975). For example, the tonotopic organization in auditory cortex appears to be preserved under a variety of anesthetic agents (Merzenich et al., 1975; Guo et al., 2012). Dose-dependent suppression by isoflurane of some, but not all, epidurally-recorded evoked responses has been observed (Santarelli et al., 2003), though in visual cortex high anesthetic doses have been reported to enhance LFP responses to sensory stimuli (Imas et al., 2005b). We observed similar enhanced response amplitude at the highest dose (1.6%) tested, which was sufficient to cause a burst-suppression spontaneous activity pattern (not shown) (Hartikainen et al., 1995). At this concentration, both auditory and visual stimuli could elicit burst responses that were stereotypical and likely reflected engagement of the same cortical circuitry underlying spontaneous bursts. At all concentrations tested, however, the magnitude and overall pattern of the response to auditory stimulation was relatively resistant to isoflurane (Figures 4, 5). In brain slices, isoflurane suppressed early current sinks in response to TC stimulation, though the effect was much more modest than for CC responses (Figures 7, 8). It is possible that enhanced acoustic responses in the auditory periphery compensate *in vivo* for suppression of TC synaptic responses in cortex; alternatively, species differences may account for some of the differences between our results *in vivo* and in slices (see above).

Our results *in vivo* stand in contrast to the thalamic switch hypothesis (Alkire et al., 2000), in which anesthetics cause LOC by impairing information flow along the TC pathway. Several studies have reported suppressed activity in thalamus at clinically relevant doses, both *in vitro* and *in vivo* (Ries and Puil, 1999; Alkire et al., 2000; Alkire, 2008; Langsjo et al., 2012; Schroter et al., 2012), and thalamic micro-injections of GABAergic and cholinergic agonists can trigger loss and recovery of consciousness, respectively, in rats (Miller, 1992; Alkire et al., 2007). However, evidence suggests that anesthetics selectively target non-specific thalamic nuclei, leaving ascending sensory pathways intact (Liu et al., 2013), and, as for anesthetic effects on cortical activity, reductions in thalamic activity can occur even when LOC does not (Alkire et al., 2008a).

In contrast to relatively preserved signals in sensory cortex for stimuli of the primary modality and for core TC responses, we have shown that responses to stimuli of a secondary modality and CC responses are preferentially suppressed by isoflurane. These results are broadly supportive of a mechanism based on cortico-thalamic network disruption, derived from the information integration theory of consciousness and the cognitive unbinding hypothesis (Tononi, 2004; Mashour, 2013). We note that disruption of multimodal integration while leaving primary sensory pathways intact is a specific prediction of the latter model. Consistent with these results, multimodal interactions between auditory and visual cortex in humans under resting state conditions, presumably reflecting suppression of concurrent activity in the two regions that arises due to direct or indirect synaptic connections, are suppressed at hypnotic doses of propofol (Boveroux et al., 2010). Although this is the first direct observation of differential effects of anesthetics on sensory afferent pathways, these results are consistent with previous studies showing that anesthetics at hypnotic doses reduce effective connectivity between cortical areas, and especially descending connections (Ku et al., 2011; Schrouff et al., 2011; Lee et al., 2013b).

Interestingly, we observed that suppression of the visual response in auditory cortex *in vivo* reached a significant level even at sub-hypnotic doses of isoflurane. At these doses, the animal had intact righting reflex, but their overall behavior and behavioral responses to external stimuli were not assayed explicitly. The decreased response may be related to a gradual decrease in the consciousness level of the animal, or to a decreased level of sensory integration and awareness. Previous studies have shown that sub-hypnotic doses of volatile anesthetics can modulate neuronal activity (Antkowiak and Helfrichforster, 1998; Hentschke et al., 2005; Becker et al., 2012) as well as learning and a variety of behaviors (Cook et al., 1978; Dwyer et al., 1992; Alkire and Gorski, 2004; Burlingame et al., 2007). In predictive coding models, descending signals reflect memory traces engaged to predict observed responses throughout the cortical hierarchy (Rao and Ballard, 1999; Bar, 2009; Bastos et al., 2012; Wacongne et al., 2012). Evidence indicates that memory formation is extremely sensitive to anesthesia, with concentrations suppressing recall approximately one half those causing LOC (Alkire and Gorski, 2004; Perouansky et al., 2010). In humans, the incidence of recall under anesthesia is exceedingly low, estimated to occur in at most 0.1–0.2% of patients (Myles et al., 2004; Avidan et al., 2011). It is possible that this high sensitivity of memory to anesthesia is related to suppression of multimodal and CC responses (Newton et al., 1990; Alkire and Gorski, 2004).

There are a number of possible mechanisms for the differential effects of isoflurane on V2/L5 and L1 vs. core TC synaptic responses. First, isoflurane may act presynaptically, reducing neurotransmitter release in a synapse-specific way. There is precedence for this type of specificity, in that volatile agents have been shown to preferentially reduce synaptic release of glutamate compared to GABA (Perouansky et al., 1995; Maciver et al., 1996; Maciver, 1997; Kirson et al., 1998; Westphalen and Hemmings, 2006a,b; Peters et al., 2008). We note, however, that we were unable to detect evidence for a presynaptic mechanism based on paired pulse facilitation of TC, V2/L5, and L1 responses (Figure 10). Isoflurane could also affect axonal excitability, and thus sensitivity to electrical stimulation, which would not be manifest in changes in short-term plasticity. We did not observe fiber volley components in our recordings except at higher stimulus intensities than those employed here and thus we cannot exclude this possibility. However, fiber volleys in hippocampus have been shown to be insensitive to isoflurane except at extremely high concentrations (Winegar and Maciver, 2006). These observations suggest that the differential effect of isoflurane may rely on postsynaptic differences in synapse location, in which more distal synapses (V2/L5 and L1) will be more affected compared to more proximal synapses (core TC) due to anesthetic effects on postsynaptic membrane properties [e.g., potassium leak currents (Franks and Lieb, 1999; Patel et al., 1999; Putzke et al., 2007)].

### Functional implications

In predictive coding models of sensory processing, the nervous system compares at each moment in time the expectations about impending sensory input with what is actually observed (Hawkins and Blakeslee, 2005; Bar, 2009; Bastos et al., 2012). These expectations are based on memory and the statistical regularity of the physical world, and this integration of top-down and bottom-up information streams is postulated to be the critical step in sensory awareness. We have used sensory stimuli and electrical stimulation to activate selectively ascending and descending pathways to auditory cortex, and have demonstrated that descending pathways are preferentially suppressed by clinically relevant doses of the volatile anesthetic isoflurane. These data are thus consistent with the emerging model of how loss and recovery of consciousness occur under anesthesia, in which anesthetic agents preferentially suppressing top-down connections and thus interfering with predictive coding, and provide evidence that the integration of top-down and bottom-up signals is indeed a necessary component of consciousness. We note that the data presented here, although representing a direct test of the predictions of the cognitive unbinding and information integration theories, are by themselves correlative in nature. In particular, although we used doses of isoflurane calibrated to be just-hypnotic, we did not assay the specific involvement of top-down projections in consciousness, test the role of their disruption in LOC, or assay sensory awareness or recall directly. Such direct tests await future studies in which level of consciousness can be measured simultaneously with measurements of top-down connectivity, and more importantly these top-down connections can be manipulated independently to investigate their causal role in loss and recovery of consciousness. For example, experiments in which opto- or pharmacogenetic methods are used to selectively inhibit, under awake conditions, or activate, under LOC, descending and matrix thalamic projections will allow us to more firmly establish a causal role for top-down connectivity in sensory awareness.

### Conflict of interest statement

The authors declare that the research was conducted in the absence of any commercial or financial relationships that could be construed as a potential conflict of interest.
